# Why Employees (Still) Click on Phishing Links: Investigation in Hospitals

**DOI:** 10.2196/16775

**Published:** 2020-01-23

**Authors:** Mohammad S Jalali, Maike Bruckes, Daniel Westmattelmann, Gerhard Schewe

**Affiliations:** 1 Massachusetts General Hospital Institute for Technology Assessment Harvard Medical School Boston, MA United States; 2 Massachusetts Institute of Technology Sloan School of Management Cambridge, MA United States; 3 Center for Management University of Muenster Muenster Germany

**Keywords:** information security management, phishing emails, compliance, trust, theory of planned behavior

## Abstract

**Background:**

Hospitals have been one of the major targets for phishing attacks. Despite efforts to improve information security compliance, hospitals still significantly suffer from such attacks, impacting the quality of care and the safety of patients.

**Objective:**

This study aimed to investigate why hospital employees decide to click on phishing emails by analyzing actual clicking data.

**Methods:**

We first gauged the factors that influence clicking behavior using the theory of planned behavior (TPB) and integrating trust theories. We then conducted a survey in hospitals and used structural equation modeling to investigate the components of compliance intention. We matched employees’ survey results with their actual clicking data from phishing campaigns.

**Results:**

Our analysis (N=397) reveals that TPB factors (attitude, subjective norms, and perceived behavioral control), as well as collective felt trust and trust in information security technology, are positively related to compliance intention. However, compliance intention is not significantly related to compliance behavior. Only the level of employees’ workload is positively associated with the likelihood of employees clicking on a phishing link.

**Conclusions:**

This is one of the few studies in information security and decision making that observed compliance behavior by analyzing clicking data rather than using self-reported data. We show that, in the context of phishing emails, intention and compliance might not be as strongly linked as previously assumed; hence, hospitals must remain vigilant with vulnerabilities that cannot be easily managed. Importantly, given the significant association between workload and noncompliance behavior (ie, clicking on phishing links), hospitals should better manage employees’ workload to increase information security. Our findings can help health care organizations augment employees’ compliance with their cybersecurity policies and reduce the likelihood of clicking on phishing links.

## Introduction

### Background

The digitalization of health records is vastly transforming the health care industry, establishing enhanced treatment results and medical care experiences. By providing and sharing information, digital health care information systems (IS) are beneficial in various ways: they result in less chance for human error, continuous and autonomous monitoring of the patient, and more efficiency [1]. However, the increasingly complex digital systems have also resulted in major security challenges. Health care organizations are especially vulnerable to information security threats, as data breaches can have direct and severe consequences on patients’ lives [2-4]. Attacks against hospitals have been increasing in both number and level of sophistication [5].

Cybersecurity pertains to protecting internet networks and their confidential information from unwanted invasions and accidental leaks [6]. In information security management, humans are the weakest link—any employee who violates information security policies (ISPs) makes their organization vulnerable to a cybersecurity attack [7,8]. Discovering “why” employees fail to comply with ISP is critical in protecting an organization’s information.

Phishing emails demonstrate this issue. Phishing is the practice of sending emails claiming a false identity to induce individuals to reveal information. These fraudulent emails are tailored to access information systems by targeting those with access to the system. Phishing poses a major cybersecurity risk for 2 reasons: (1) employees usually have detailed knowledge about IS within the organization and access the data frequently during their work and (2) even 1 innocent click could expose the organization to a network of hackers nearly impossible to trace [9-12]. A recent study analyzed phishing campaigns in health care organizations and found that, on average, as much as 14.2% of these phishing emails were clicked on by employees [5]. Organizations have taken steps to address this problem by providing training programs to educate and increase cybersecurity awareness, but these efforts remain insufficient. In fact, research shows that mandatory training programs did not make a large difference on reducing clicking rates on phishing links [13]. Recent evidence indicates that approximately 70% of hospitals fail to establish or uphold sufficient privacy and security measures [14].

To investigate employee’s compliance with ISP, previous research has often focused on cognitive beliefs based on the theory of planned behavior (TPB) [8,9]. TPB has often been validated and is the most commonly used theory to measure different antecedents to ISP compliance [15-18]. However, previous studies have not adequately investigated the components of these cognitive beliefs. One such component is trust. Trust influences how individuals assess cost-benefit considerations and make decisions, and ultimately their behavior [19,20]. Trust has been investigated from a broad range of research directions and has evolved to a widely accepted and established concept [21-24].

Particularly in regard to phishing attacks, 2 major questions remain unanswered: (1) What is the role of trust in predicting employees’ compliance intention? and (2) To what extent does compliance *intention* correspond to compliance *behavior*? To address these questions, we drew on the TPB and investigated factors that motivate compliance with information security guidelines. We conducted a survey and used data from phishing campaigns to highlight relationships among employees’ attitudes and beliefs and their actual compliance behavior.

The study consists of 2 steps: First, as a part of phishing tests, employees of hospitals received a faux phishing email. Second, about 6 weeks apart, all individuals (clickers and nonclickers) answered a survey that examined their attitudes and positions toward cybersecurity policy. As we were comparing individuals’ qualitative answers in the survey against their own clicking data, we were able to observe and compare their compliance intention with their actual behavior.

This paper is organized as follows: We first present the theoretical background and the research model. Next, we present our research methods, including the structure of the phishing ploy and the survey. Finally, we present our data analysis, results, and discussion.

### Theory of Planned Behavior

TPB has emerged as one of the most influential frameworks for the explanation of human behavior [25,26]. The TPB explains that attitudes, subjective norms, and perception of behavioral controls (see Ajzen [27] for the definitions of these elements) form an individual’s intention to perform a certain behavior—intention is a direct antecedent of the actual behavior. A positive attitude toward ISP is assumed to predict compliance intention. Bulgurcu et al [9] focus on the link between employees’ attitudes toward compliance and their intention to comply and found a positive relationship. Similarly, Ifinedo [28] investigated ISP compliance of managers and IS professionals. These studies concluded that attitude toward compliance, subjective norms, and response efficacy positively influence employees’ general ISP compliance intentions. Although these findings all show that TPB is generally suitable for predicting intention in information security research, the specific context (ie, phishing) is a major influence on the behavioral intention—as the context might substantially influence the outcome. Thus, we build on previous research by proposing that TPB variables are associated with employees’ intention to comply specifically with ISP:

H1a: Attitudes toward ISP is positively related to the intention to comply.

H1b: Subjective norm is positively related to the intention to comply.

H1c: Perceived behavioral controls are positively related to the intention to comply.

H2: The intention to comply is positively related to compliance behavior.

### Collective Felt Trust

A second factor we believe influences compliance is collective felt trust. In their review, Mayer et al [29] suggest that trust influences employees’ behavior in the sense that it affects risk-taking in relationships and impacts processes and outcomes in an organization. Trust is defined as “a psychological state comprising the intention to accept vulnerability based upon positive expectations of the intentions or behavior of another” [24].

Trust has previously been shown to influence attitudes in the TPB. Pavlou et al [30] investigated whether trust is relevant for the attitude toward a certain product. They found that trust in the person providing a product had a significant effect on attitude toward the product. Management of an organization is responsible for providing a work environment within the company that enables employees to focus on their tasks. Moreover, trust has been shown to impact organizational support and commitment [31,32] and organizational citizenship behavior [33,34]. Meta-analytic evidence has shown that by trusting the management, employees feel more committed to their company and will be more willing to follow organizational policies [35]. Several studies report a positive relationship between trust and compliance [36-39]. In addition to this relationship, Deutsch Salamon and Robinson [40] found that felt trust increased employees’ responsibility norms and subsequently their performance. We assume that this effect holds in this context too: Employees that feel trusted by their management consider their behavior more closely to not violate the trust they are being given.

On the basis of these considerations, we argue that collective felt trust influences employees’ attitudes toward ISP and their perceived subjective norms. Thus, we propose:

H3a: Collective felt trust is positively related to the attitudes toward ISP.

H3b: Collective felt trust is positively related to subjective norms.

### Trust in Technology

Although trust has often been researched on the interpersonal level, recent developments show that trust in technology is equally important [41,42]. Trust in technology has been shown to increase the acceptance and intention to use technologies [41,43], such as information and technology (IT) artifacts [44] and cloud technologies [45].

When individuals find themselves in risky situations in which they have to depend on technologies, trust in technology becomes essential [46]. Individuals are sensitive to the functioning of that specific technology—similar to trust in people, trust in technology is formed based on the perception of the attributes of technology. Lankton et al [22] suggest differentiating among perceptions of functionality, helpfulness, and reliability as factors affecting trust in technology. In the context of information security, the helpfulness (eg, of an antivirus) is rather limited, although functionality and reliability are highly relevant. High trust in technology will enhance the level of confidence in facing cyber threats. We therefore assume that:

H4: Trust in technology, consisting of (1) reliability and (2) functionality, is positively related to perceived behavioral control.

In situations where individuals perceive a higher threat of cyberattacks, the attention toward potential harms might rise. Johnston et al [11] discuss the influence of fear appeals in IS security and argue that the more severe or susceptible a threat is perceived to be, the fewer individuals rely on the ability of the cybersecurity software. Thus, the higher the perceived risk, the more individuals are expected to pay attention to situations where the software did not adequately eliminate the threat, that is, phishing emails. We therefore propose that:

H5: Perceived risk is positively related to the compliance behavior.

On the other side, employees that face a high workload might not be able to execute cognitive considerations to decide to follow ISP. Employees might even use their high workload as an excuse for violating ISP [12]. In situations where high workload stops employees from paying attention to details of an email, whether intentionally or accidentally, the likelihood of opening a potentially dangerous email might increase. Thus, we propose that:

H6: High workload is negatively related to the compliance behavior.

**Figure 1:**
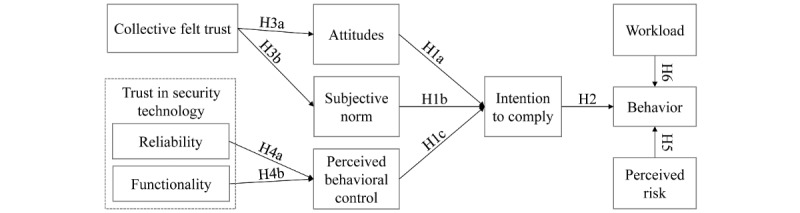
Proposed research model. H: hypothesis.

Putting these hypotheses together, Figure 1 presents our proposed research model.

## Methods

### Data and Procedure

Data were collected in 2 steps. In the first step, a professional cybersecurity company sent out phishing emails to employees in 3 networks of hospitals in the eastern United States. The phishing campaigns were designed to resemble real phishing emails so that participants could not know that they are being tested and would behave as if they received a real phishing email. All phishing emails contained a hyperlink. Collected data included the identity of individuals receiving the email and whether they clicked on the link or not. This information was then provided only to the respective hospital.

For the second step, we developed a Web-based survey instrument. To compare the results of clickers and nonclickers, we created 2 different survey links based on the same questionnaire. The key constructs with the underlying items are listed in Table 1. 

**Table 1:** Survey items.

Construct and items		Loadings	Cronbach alpha
**Attitudes toward information security policy**			.86
	I believe it is beneficial for our organization to establish clear information security policies, practices, and technologies.^a^	0.891	
	I believe it is useful for our organization to enforce its information security policies, practices, and technologies.^a^	0.756	
	I believe it is a good idea for our organization to establish clear information security policies, practices, and technologies.^a^	0.884	
**Subjective norm**			.93
	People who influenced my behavior would think that I should follow the policies and procedures and use the cybersecurity technologies.^a^	0.844	
	People whose opinions are important to me would think that I should follow the policies and procedures and use the cybersecurity technologies.^a^	0.955	
	People whom I respect would think that I should follow the policies and procedures and use the cybersecurity technologies.^a^	0.952	
**Perceived behavioral control**			.79
	I am able to follow the cybersecurity policies and procedures and technologies (eg, antivirus, or other products).^a^	0.665	
	I have the resources and knowledge to follow the policies and procedures and use the cybersecurity technologies.^a^	0.917	
	I have adequate training to follow the policies and procedures and use cybersecurity technologies.^a^	0.850	
**Intention to comply**			1
	I intend to follow the information security policies and practices at work.^c^	1	
**Collective felt trust**			.77
	Management lets me have an impact on issues they find important.^a^	Dropped	
	Management does not feel the need to *keep an eye* on me.^a^	0.773	
	Management would be comfortable assigning me a critical task, even if they cannot monitor my actions.^a^	0.735	
	Management believes that employees can be trusted.^a^	0.688	
**Trust in technology—reliability**			.95
	The cybersecurity software at my workplace (eg, antivirus and firewall) is reliable.^a^	0.897	
	The cybersecurity software at my workplace does not fail me.^a^	0.939	
	The cybersecurity software at my workplace provides accurate service.^a^	0.893	
**Trust in technology—functionality**			.95
	The cybersecurity software at my workplace has the functionality I need.^a^	0.946	
	The cybersecurity software at my workplace has the features required for my tasks.^a^	0.929	
	The cybersecurity software at my workplace has the ability to do what I want it to do.^a^	0.909	
**Perceived information security risk**			.93
	At my workplace, the risk to my computer and data from Internet security breaches is^d^:	0.704	
	At my workplace, the likelihood that my computer will be disrupted due to Internet security breaches within the next 12 months is^d^:	0.918	
	At my workplace, the chance that my computer will fall a victim to an Internet security breach is^d^:	0.967	
	At my workplace, the vulnerability of my computer and data to Internet security risks is^d^:	0.910	
**Workload**			.82
	I feel that the number of requests, problems, or complaints I deal with at work is more than expected.^a^	Dropped	
	I feel that the amount of work I do interferes with how well it is done.^a^	0.588	
	I feel busy or rushed at work. (R)^e^	0.916	
	I feel pressured at work. (R)^e^	0.818	

^a^Strongly agree, somewhat agree, neither agree nor disagree, somewhat disagree, strongly disagree.

^b^Not applicable.

^c^Single-item measurement; strongly agree, agree, somewhat agree, neither agree nor disagree, somewhat disagree, disagree, strongly disagree.

^d^Extremely high, somewhat high, neither high nor low, somewhat low, or extremely low.

^e^(R): Reverse coded item; always, most of the time, about half the time, sometimes, never.

As hospitals’ IT departments knew the identity of clickers, they distributed 1 survey link to employees that had clicked on the phishing link and the other survey link to those that had not. This separation helped facilitate the anonymity of the survey analysis, as we did not ask hospitals for any clicking data. Participants were informed that their participation in our survey was voluntary and anonymous.

By combining these 2 steps, we aimed to independently and systematically assess the extent to which attitudes and attributes are related to the actual clicking behavior. Collecting clicking data in the first step has the advantage that the results are not distorted by the survey. To nullify the concern of whether having clicked or not clicked on the phishing email would influence behavior in the survey, the survey was distributed about 6 weeks after the phishing emails were sent out.

### Measures

The survey contained questions about the personal attitudes toward the company and its ISP. A pilot test was run with 10 researchers to ensure that all questions were clear. A 5-point Likert scale (1=never, strongly disagree, or extremely low; and 5=always, strongly agree, or extremely high) was used for all items except for intention, our only single-item measurement, where a 7-point Likert scale (1=strongly disagree and 7=strongly agree) was used. See recommendations by Fuchs and Diamantopoulos [47] and Wirtz and Lee [48] for using a larger scale for single-item measurements.

All survey items were based on previously validated items to maximize reliability. The 9 constructs of the survey include attitudes, subjective norm, perceived behavioral control, intention [8,25], collective felt trust [29,40], trust in security technology based on reliability and functionality [22,41], perceived security risk [49], and workload [50].

As control variables in addition to the core concepts, we also asked for the average number of emails received daily, age, position (clinical or nonclinical), and education level.

### Data Analysis

The survey was sent to 3169 employees in 3 hospital networks. A total of 488 individuals participated in the study (488/3169, 15.40% response rate). Owing to missing data in variables essential for the proposed research model (eg, *Intention to comply*), 58 participants were excluded from the analysis. To minimize external influences, we also excluded participants from hospital network C, because from this hospital (a small local hospital), only 33 employees participated in the survey. Together, this led to the exclusion of 91 participants and a final sample of 397 (397/3100, 12.80% overall response rate). Of the remaining participants, 172 were from hospital network A, and 225 were from hospital network B.

Table 2 presents the individual response rates, and Table 3 presents the respondent characteristics of the final sample. The respondent characteristics show that the sample is heterogeneous, having a positive impact on the external validity of this study (see Table 3).

As a proxy to test for nonresponse bias, we followed the recommendations by Armstrong and Overton [51] and tested differences in age, gender, position, education, and clicking behavior between early and late respondents. *t* test results show no significant differences between these 2 groups.

To test the strength of the relationship among different constructs and its effect on the actual clicking behavior, we used partial least squared structural equation modeling (SEM) in software SmartPLS (SmartPLS GmbH). PLS was chosen over covariance-based SEM, as it is widely applied in information security research [52] and does not assume a normal distribution, is particularly appropriate for complex models, and its bootstrapping method increases robustness [53].

Before testing the SEM, we assessed the constructs’ loadings and Cronbach alphas to evaluate the reliability of the measurement model. After 2 items were dropped from the analysis, all loadings were above the common threshold value of 0.70 [53]. In addition, Cronbach alphas all exceeded .70, also indicating good reliability [53]. Furthermore, the constructs showed adequate convergent validity as the average variance extracted (AVE) was above 0.68 and composite reliability was above 0.70 for all factors.

Discriminant validity was tested by using the Fornell-Lacker Criterion and Heterotrait-Monotrait ratios. The Fornell-Lacker Criterion indicated that the square root of the AVE of each construct was higher than the construct’s correlation with any other construct [54]. In addition, Heterotrait-Monotrait ratios were below the threshold of 0.9, also confirming discriminant validity for the measurement models [55]. Finally, all variance inflation factors values were below 5, which suggests multicollinearity is unlikely to be a problem [53]. The relevant reliability and validity fit indices on construct level are reported in Table 4.

**Table 2:** Response rates.

Hospital network and target group		Employees who received the questionnaire (N)	Responses included in the analysis, n (%)
**Hospital network A**			
	Total	2100	172 (8.20)
	Clicker	1600	122 (7.63)
	Nonclicker	500	50 (10.0)
**Hospital network B**			
	Total	1000	225 (22.50)
	Clicker	500	109 (21.8)
	Nonclicker	500	116 (23.2)
Overall sample total		3100	397 (12.80)

**Table 3:** Respondent characteristics (N=397).

Category		Count, n (%)
**Sex**		
	Male	82 (22.09)
	Female	309 (76.28)
	Nonbinary	2 (0.47)
	Unanswered	4 (1.16)
**Age (years)**		
	18-24	28 (7.05)
	25-34	108 (27.20)
	35-44	70 (17.63)
	45-54	78 (19.65)
	55-64	86 (21.66)
	65-74	19 (4.79)
	≥75	2 (0.50)
	Unanswered	6 (1.51)
**Position**		
	Clinical	221 (55.67)
	Nonclinical	172 (43.32)
	Unanswered	4 (1.01)
**Education**		
	Less than high school	28 (7.30)
	High school graduate	47 (11.84)
	Some college	111 (27.96)
	2-year degree	43 (10.83)
	4-year degree	120 (30.23)
	Professional degree	41 (10.33)
	Unanswered	6 (1.51)
**Emails per day**		
	<10	87 (21.91)
	11-20	133 (33.50)
	21-30	72 (18.14)
	>31	101 (25.44)
	Unanswered	4 (1.01)
**Response to phishing email**		
	Clicker	231 (58.19)
	Nonclicker	166 (41.81)

**Table 4:** Reliability and validity of measurement model.

Construct	Cronbach alpha	Average variance extracted	Composite reliability	Heterotrait-Monotrait ratio							
				Attitudes	Subjective norm	Perceived behavioral control	Intention to comply	Collective felt trust	Reliability	Functionality	Perceived risk
Attitudes	.88	0.80	0.92	—^a^	—	—	—	—	—	—	—
Subjective norm	.94	0.89	0.96	0.391	—	—	—	—	—	—	—
Perceived behavioral control	.84	0.76	0.90	0.419	0.381	—	—	—	—	—	—
Intention to comply	N/A^b^	N/A	N/A	0.486	0.337	0.621	—	—	—	—	—
Collective felt trust	.76	0.69	0.87	0.270	0.208	0.270	0.289	—	—	—	—
Reliability	.94	0.89	0.96	0.298	0.251	0.320	0.466	0.324	—	—	—
Functionality	.95	0.91	0.97	0.289	0.231	0.510	0.382	0.351	0.871	—	—
Perceived risk	.93	0.83	0.95	0.117	0.165	0.252	0.270	0.299	0.320	0.196	—
Workload	.81	0.73	0.89	0.122	0.032	0.224	0.146	0.219	0.161	0.188	0.178

^a^Table is symmetric, only the lower triangle is presented.

^b^N/A: not applicable.

## Results

**Figure 2:**
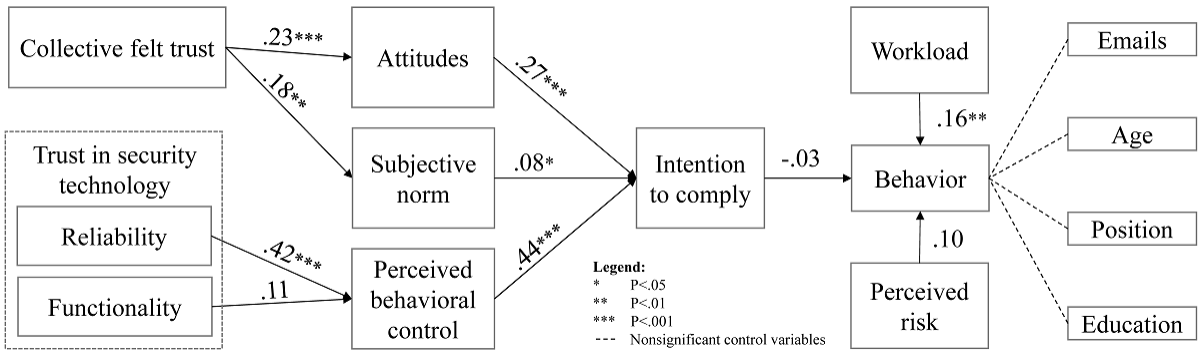
Results of structural equation model.

Table 5 reports means, SDs, and zero-order correlations of all latent variables. For testing the SEM, bias-corrected bootstrapping based on a bootstrap sample of 5000 was applied [53]. The standardized paths coefficients and significance levels are presented in Figure 2.

Hypothesis 1 predicted that (1) attitudes toward ISP, (2) subjective norms, and (3) perceived behavioral control are positively related to the intention to comply. This prediction is supported: attitudes toward ISP (beta=.27; *P*<.001), subjective norm (beta=.08; *P*=.04), and perceived behavioral control (beta=.44; *P*<.001) showed significant relationships with intention to comply.

Hypothesis 2 predicted that the intention to comply is positively related to compliance behavior. Contrary to the assumption, our results show that intention and clicking behavior are not significantly related in our analysis (beta=−.03; *P*=.57). Thus, hypothesis 2 is not supported.

Hypothesis 3 predicted that collective felt trust is positively related to attitudes toward ISP and subjective norms. Our results support this hypothesis: Trust is significantly related to attitudes toward ISP (beta=.23; *P*<.001) and subjective norm (beta=.18; *P*=.001).

Hypothesis 4 predicted that trust in security technology, consisting of (1) reliability and (2) functionality, is positively related to perceived behavioral control. Our results support hypothesis 4a (beta=.42; *P*<.001) but not 4b (beta=.11; *P*=.15). Thus, trust in security technology is solely based on reliability perceptions.

Hypothesis 5 predicted that a higher perceived risk of cyberattacks is negatively related to the likelihood to click on phishing links. This hypothesis cannot be supported as our results indicate no significant relationship between perceived risk and the behavior to click on phishing links (beta=.10; *P*=.05).

Finally, hypothesis 6 predicted that high workload is positively related to the likelihood of clicking on phishing links. Our results show that this relationship is indeed significant, supporting this hypothesis (beta=.16; *P*=.001).

We included several control variables to test whether clicking on a phishing link was different for age groups, education levels, positions (clinical or nonclinical), or the number of emails received per day. None of these variables had a significant effect on the behavior of clicking on the link in the phishing email.

**Table 5:** Zero-order correlations and descriptive statistics.

Construct	Value, mean (SD)	Zero-order correlations							
		Attitudes	Subjective norm	Perceived behavioral control	Intention to comply	Collective felt trust	Reliability	Functionality	Perceived risk
Attitudes	4.79 (0.42)	—^a^	—	—	—	—	—	—	—
Subjective norm	4.42 (0.72)	.38^b^	—	—	—	—	—	—	—
Perceived behavioral control	4.46 (0.38)	.34^b^	.34^b^	—	—	—	—	—	—
Intention to comply	6.69 (0.572)	.47^b^	.34^b^	.58^b^	—	—	—	—	—
Collective felt trust	4.81 (0.88)	.26^b^	.21^b^	.23^b^	.29^b^	—	—	—	—
Reliability	4.09 (0.75)	.28^b^	.25^b^	.55^b^	.46^b^	.32^b^	—	—	—
Functionality	4.05 (0.92)	.27^b^	.22^b^	.49^b^	.38^b^	.34^b^	.87^b^	—	—
Perceived risk	2.46 (0.84)	−.10	−.16^c^	−.24^b^	−.26^b^	−.29^b^	−.32^b^	−.20^b^	
Workload	2.76 (0.72)	−.11	.01	−.18^c^	−.12^d^	−.19^c^	−.14^d^	−.17^c^	.16^c^

^a^Table is symmetric, only the lower triangle is presented.

^b^*P*<.001, 2-tailed.

^c^*P*<.01, 2-tailed.

^d^*P*<.05, 2-tailed.

Evaluating the overall model, we find that the data fit well for intention to comply with an R² of 0.397. The Stone-Geisser test has shown with a Q² value of 0.377 that the model has a large predictive relevance for intention to comply. In contrast, the model explains little variance of the clicking behavior (R²=0.044) and has a small predictive relevance for this construct (Q²=0.040).

Employees from 2 hospital networks were included in this analysis, with 172 participants being employed by hospital network A and 225 by hospital network B. Performing a subgroup analysis, we determined whether the hospital context affected the SEM results. Results of the subgroup analysis show no group differences regarding the (non) significant paths (see Table 6). Hypothesis 1b is only partially supported, as the overall effect is significant here, although no significant effect can be identified in hospitals A and B.

A multigroup analysis (MGA) was performed to test whether any significant differences in these path coefficients exist. The test for measurement invariance shows no significant differences in the measurement models between hospital networks A and B, which indicates that potential differences are not based on measurement error and that MGA provides reliable results at the construct level. In the overall sample, no control variable has a significant effect on the clicking behavior. However, the MGA reveals that the position (clinical vs nonclinical) significantly affects the clicking behavior in hospital B and that this effect differs significantly from the effect in hospital A (|hospital A-B|=0.216; *P*>.99). On the other hand, in hospital A, education has a significantly negative effect on the clicking behavior. This effect is not observed in hospital B and is significantly different (|hospital A-B|=0.184; *P*=.99).

**Table 6:** Results of structural equation model and its multi-group analysis.

Hypotheses		Overall sample		Hospital A		Hospital B		Multigroup analysis		Assessment of hypotheses
		Beta^a^	*P* value	Beta	*P* value	Beta	*P* value	|Difference|	*P* value	
H1a		.268	<.001	.367	<.001	.172	.047	.195	.11	Supported
H1b		.083	.04	.105	.09	.053	.32	.052	.52	Partly supported
H1c		.444	<.001	.403	<.001	.490	<.001	.087	.40	Supported
H2		−.037	.45	−.041	.58	−.021	.76	.020	.85	Rejected
H3a		.229	<.001	.238	<.001	.234	<.001	.004	.97	Supported
H3b		.178	<.001	.179	.02	.178	.02	.001	.99	Supported
H4a		.421	<.001	.424	<.001	.435	<.001	.011	.95	Supported
H4b		.112	.15	.144	.20	.087	.41	.057	.71	Rejected
H5		.099	.05	.051	.58	.091	.22	.040	.73	Rejected
H6		.157	<.001	.242	<.001	.137	.04	.105	.26	Supported
**Controls**										
	Emails	.063	.26	−.071	.39	.112	.10	.183	.93	—^b^
	Age	.013	.81	−.070	.36	.027	.72	.097	.82	—^b^
	Position	.076	.14	−.083	.10	.133	<.001	.216	>.99	—^b^
	Education	.018	.74	−.108	.01	.076	.25	.184	.99	—^b^

^a^Beta=effect size.

^b^Not applicable.

## Discussion

### Principal Findings

This study investigates the relationship between employees’ compliance intention and their actual compliance with ISPs (ie, not clicking on the phishing link). As hypothesized in H1, we found that attitudes, subjective norm, and perceived behavioral control were positively related to the intention to comply with organizational ISPs. However, contrary to what was suggested in H2, there was no significant relationship between the intention to comply and the compliance behavior itself. In contrast to this finding, previous studies have provided evidence for a positive relationship [56,57]. However, because of the difficulty of observing actual behavior, these studies have relied on self-reported data to assess the relationship between intention and the actual compliance. This process leaves room for method biases because individuals could give desirable answers, or previous answers could influence later answers [58,59]. In a recent review of employees’ security behavior, the authors challenge the assumption that intention predicts behavior in the information security context [15]. In line with this, our results indicate that in the context of phishing emails, intention and compliance might not be as strongly linked as previously assumed. Thus, the role of context in compliance investigations should be carefully considered as it might prove to be highly relevant.

We also found that collective felt trust was significantly related to employees’ attitudes and subjective norms, supporting H3. Higher collective felt trust is associated with more positive attitudes and subjective norms, which in turn positively influence the compliance intention. The results indicate that management can have an influence on how employees perceive security policies. Moreover, the rich literature of trust and control points toward another benefit: Trust in the management reduces the risk that employees perceive security policies as a sign of management distrust in them and their abilities [60]—employees might understand that ISP are not designed to reduce their freedom but to enhance their protection. In addition, with high levels of trust, employees are likely to internalize the organization’s goals and thus are more willing to protect the company by accepting the policies [61,62].

Considering the relationship between trust in technology and perceived behavioral control, we found that only trust in technology based on reliability has a significant influence on an employee’s perceived behavioral control. Trust based on functionality was not significantly related. This finding supports H4a but not H4b. With high trust in information security technologies in use, employees may think that they are more capable of controlling their own behavior. Trust in technology has been shown to increase the adoption of new technologies and has frequently been used in IS literature [63,64]. Such reliance and trust in technology is also seen in health care settings as medical professions constantly introduce or utilize programs that allow most convenient—as well as easy—access to patient records [65]. This is done to ensure that physicians can offer the best care for their patients. Unbeknown to them, however, this also allows for easier access to sensitive personal information for attackers. Furthermore, research on information system security has not yet been adopted into this concept. Thus, these findings not only contribute to security compliance but also enhance the understanding of application areas for trust in technology.

Moreover, we found no significant effect of perceived risk on compliance behavior, contrary to what was assumed in H5. A reason for this might be that the risk is too abstract for employees or that the perceived benefits in a certain situation outweigh the perceived risk [49]. More specifically, in the health care setting, the risk of clicking on a foreign email or revealing sensitive information would most likely outweigh the risk of patient safety, treatment quality, private information, and data theft—which would be the most plausible explanation for this result [66].

As assumed in H6, we found a significant effect between workload and compliance behavior. As none of the cognitive variables showed a significant relationship with the behavior, the workload is the only variable related to the compliance behavior. This finding is interesting because it offers an insight into the situations in which phishing emails are opened. Any form of noncompliance behavior (in this case, the necessity to cope with a high workload) might lead to less eagerness to follow security policies [12]. Furthermore, high workload might cause unintended noncompliance behavior—high volumes of work could make one click on a phishing link because an overworked employee could have been too occupied to notice the imposed threats [15]. This is especially concerning given that cyberattacks today are extremely hard to detect because they have become extremely intricate; they are targeted attacks that have been carefully planned according to each organization’s needs [67]. Tactics such as social engineering—the act of psychologically manipulating people into revealing personal information or allowing access to a secured server—have been increasingly successful in phishing [68]. More specifically, spear-phishing, a specified type of attack, uses context-specific, sophisticated emails that are tailored to meet individual and company-specific needs. It is difficult to detect—and requires much attention to detect—because the reader must consider the *plausibility* of the written text—rather than visual or auditory deception [69]. In the case of an overworked, occupied employee, such sophisticated attacks are more likely to be successful.

### Practical Implications

Our findings offer a number of practical implications. Practitioners need to consider organizational factors when designing security policies and training programs. Our results show that engaging in trust-building activities can subsequently enhance employee’s compliance. Our findings also highlight the relevance of top management participation and imply that managers need to show that they acknowledge the problems associated with IS security and are able to provide a foundation of security policy and behavior upon which employees can build [8,70,71].

Furthermore, the positive relationship between trust in technology and perceived behavioral control indicates that the feeling of reliance on technology is associated with a higher intention to comply. Besides ensuring good quality of security technologies, managers need to communicate and inform employees about security technologies. If employees cannot learn about the technology in place, they cannot know how much to rely on it. Trust in technology can be developed through training and by enhancing understanding of the technology—see Puhakinen and Siponen [70] and Safa et al [72] for more discussion.

As our results show that in the context of phishing emails, the compliance intention was not related to the actual compliance behavior, hospitals must remain vigilant with vulnerabilities that cannot be easily managed.

Finally, our results present a relationship between workload and noncompliance behaviors. This finding suggests that hospitals should better manage workload to increase information security—for instance, extensive emailing may unnecessarily add to workload. Our observations working with organizations show that in addition to communication with colleagues through emails, individuals receive multiple emails on a daily basis including announcements and other general notes, which add to individuals’ email loads, putting them in more risks of clicking on phishing emails.

### Limitations

Although this study provides several insights, it is also subject to some limitations. First, the low response rate and the gender imbalance in our sample might indicate a selection bias. Selection bias is often associated with low generalizability of the results, as it is assumed that only a certain group of people responded to a study. Previous research has reported that response rates are generally low in this field [73,74]. To investigate potential bias that arise from this issue, we checked for nonresponse bias via factor analysis using the principal component as well as marker variable test [59,75,76]. All tests showed nonsignificant results, and although the presence of such bias can never be completely excluded, the results suggest that bias is not an issue in our analysis. We also included gender as a control variable in our model; however, the results showed no significant influence. As gender does not explain additional variance, we excluded this control variable from the analysis.

Although our results provide evidence for an insignificant relationship between intention and behavior, future research should investigate this relationship in a different context to validate these findings. We used a specific case of phishing emails to investigate employees’ compliance in hospitals. As previous studies have shown, the effects between TPB constructs and influencing variables might depend on the underlying scenario. Moody et al [17] found support for TPB in scenarios concerning USB use, workstation logout, and password sharing. The intention-behavior gap might be more relevant in certain situations than in others. For instance, employees might not intend to open a suspicious email but then end up doing it because of spontaneous curiosity or inattentiveness. Moreover, we focused on the hospital industry to keep the sample as consistent as possible. This restriction might limit the generalizability of our results. Organizational factors and governance structure should be considered when transmitting these results to other industry settings.

Finally, we used a generic measure to assess the intention to comply with ISP, which means we asked for general compliance instead of focusing on phishing emails. We did so because (1) we were interested in the general assessment of their own intention to comply and (2) we did not want to influence the response by drawing attention toward phishing specifically. We think that a generic measure is justified in this situation because employees in the investigated hospitals are expected to know about phishing email regulations. In both hospital networks we investigated, information security staff had already raised awareness of this issue among the employees—both hospital networks had antiphishing email training. Therefore, we did not want to draw additional attention to this matter but pose a broader question about general compliance.

### Conclusions

Employees’ compliance with policies is a key concern in information security research, especially because even an accidental security breach from a phishing attack could severely impair sensitive patient information and safety in a health care setting. This study focused on factors related to employees’ compliance intention and their actual compliance (ie, not clicking on phishing links) in hospitals. Using the lens of the TPB, we investigated the role of collective felt trust and trust in technology as an influence on attitudes, subjective norms, and perceived behavioral control. We found positive effects between collective felt trust and attitudes toward compliance and subjective norms. Trust in technology was strongly related to perceived behavioral control. In the health care setting, this trust is even more evident and exploited, as previous research revealed that more than half of the information breaches came from within the health care organizations. In addition, employees showed strong preference for trust in electronic records—over paper records—because online systems were easily accessible remotely or offsite and readily available in cases of emergency [77].

Surprisingly, we did not find an association between the intention to comply and the actual compliance behavior. With major improvements in cyberattack technologies (ie, tailoring information to be specific to the needs of the target audience or organization), it becomes nearly impossible for both employees and servers to filter phishing emails, and so employees with high intention to comply may still fall in the trap of the hackers. However, we found that a higher workload was positively related to noncompliance behavior. This finding suggests that, in the context of phishing emails, context effects are highly relevant.

A major strength of this study is that we separated data collection for the dependent (clicking behavior) and the independent (personal and organizational) variables. This is one of the few studies in information security literature that observed the compliance behavior rather than using self-reported data. This approach enabled us to obtain more reliable outcomes, given that self-reports may differ based on perception and mood, among others. We hope that our findings motivate the information security community to improve current training programs and design effective interventions to increase information security compliance.

## Abbreviations

AVE: average variance extracted
IS: information systems
ISP: information security policies
IT: information and technology
SEM: structural equation modeling
TPB: theory of planned behavior
